# *stim2b* Knockout Induces Hyperactivity and Susceptibility to Seizures in Zebrafish Larvae

**DOI:** 10.3390/cells9051285

**Published:** 2020-05-21

**Authors:** Iga Wasilewska, Rishikesh Kumar Gupta, Bartosz Wojtaś, Oksana Palchevska, Jacek Kuźnicki

**Affiliations:** 1 International Institute of Molecular and Cell Biology, 4 Ks. Trojdena Street, 02-109 Warsaw, Poland; iwasilewska@iimcb.gov.pl (I.W.); rkgupta@iimcb.gov.pl (R.K.G.); opalchevska@iimcb.gov.pl (O.P.); 2 Nencki Institute of Experimental Biology, Polish Academy of Sciences, 3 Pasteur Street, 02-093 Warsaw, Poland; b.wojtas@nencki.edu.pl

**Keywords:** Stim2b, zebrafish, behavior, in vivo imaging, calcium, GCaMP5G, seizures

## Abstract

In neurons, stromal interaction molecule (STIM) proteins regulate store-operated Ca^2+^ entry (SOCE) and are involved in calcium signaling pathways. However, STIM activity in neurological diseases is unclear and should be clarified by studies that are performed in vivo rather than in cultured cells in vitro. The present study investigated the role of neuronal Stim2b protein in zebrafish. We generated *stim2b* knockout zebrafish, which were fertile and had a regular lifespan. Using various behavioral tests, we found that *stim2b^−/−^* zebrafish larvae were hyperactive compared with wild-type fish. The mutants exhibited increases in mobility and thigmotaxis and disruptions of phototaxis. They were also more sensitive to pentylenetetrazol and glutamate treatments. Using lightsheet microscopy, a higher average oscillation frequency and higher average amplitude of neuronal Ca^2+^ oscillations were observed in *stim2b^−/−^* larvae. RNA sequencing detected upregulation of the *annexin 3a* and *gpr39* genes and downregulation of the *rrm2*, *neuroguidin*, and *homer2* genes. The latter gene encodes a protein that is involved in several processes that are involved in Ca^2+^ homeostasis in neurons, including metabotropic glutamate receptors. We propose that Stim2b deficiency in neurons dysregulates SOCE and triggers changes in gene expression, thereby causing abnormal behavior, such as hyperactivity and susceptibility to seizures.

## 1. Introduction

Stromal interaction molecule (STIM) is a calcium (Ca^2+^)-sensing protein that is localized in the endoplasmic reticulum (ER) membrane [1]. In the luminal part, it contains a highly conserved Ca^2+^-binding EF-hand domain. The cytosolic part contains the CRAC activation domain (CAD; also known as the STIM1 Orai1 activating region [SOAR]), which is responsible for interactions with plasma membrane (PM) Ca^2+^ channels and communication with calmodulin [2]. Mammals express two paralogues of STIM: *Stim1* and *Stim2*, while in fish, an additional genome duplication resulted in the appearance of two additional genes that encode Stim proteins: *stim1a*, *stim1b*, *stim2a*, and *stim2b* [3,4]. Both STIM1 and STIM2 were found to be ubiquitously expressed with varying STIM1-to-STIM2 ratios, depending on the specific tissue. In mice, STIM1 expression dominates over STIM2 expression. One exception is in the brain, mostly in the hippocampus and cortex, where higher amounts of STIM2 mRNA and protein expression have been observed [5,6,7]. In the human brain, the expression of both STIM1 and STIM2 was highest in the cortex, caudate, and hippocampus [6]. The main function of STIMs is the regulation of store-operated Ca^2+^ entry (SOCE), which enables the influx of Ca^2+^ from the external milieu into the cell under conditions of ER depletion. Upon interactions with STIM proteins, Ca^2+^ enters the cell via Orai channels that are highly selective for these ions or via less selective transient receptor potential channels [8]. The ER is then refilled with Ca^2+^ by the sarcoendoplasmic reticulum Ca^2+^ adenosine triphosophatase (SERCA) pump. The current model of SOCE considers different properties of STIM isoforms and proposes a biphasic response. Small decreases in Ca^2+^ levels in the ER are detected by STIM2, which is more sensitive to changes in Ca^2+^ levels and induces small but prolonged SOCE. STIM1 is activated by larger decreases in ER Ca^2+^ levels and triggers robust and rapid Ca^2+^ influx [2,9]. Notably, refilling the ER with Ca^2+^ is not the only consequence of SOCE. Ca^2+^ that enters the cell is involved in different signaling pathways. One of the most studied pathways is the activation of gene transcription via the calcineurin-nuclear factor of activated T-cells (NFAT) pathway (reviewed in [9,10]).

Research on the role of SOCE in neurons was relatively neglected for quite a long time. The regulation of Ca^2+^ signaling in these cells is very complex and requires tight spatial and temporal control because these ions are crucial for various neuronal processes, including neurotransmitter release, synaptic plasticity, neurogenesis, and gene transcription. Thus, neurons developed highly complicated Ca^2+^ machinery [11]. The main pathways that enable the influx of Ca^2+^ into these cells involve Ca^2+^-permeable voltage-operated Ca^2+^ channels (VOCCs) or receptor-operated channels (ROCs), such as glutamate-sensitive *N*-methyl-D-aspartate receptors (NMDARs). Nevertheless, SOCE has also been observed in neurons (reviewed in [9,10,12]). Our group demonstrated that the overexpression of STIM1 together with Orai1 increased Ca^2+^ entry that was induced by ER depletion with the potent SERCA inhibitor thapsigargin in cortical rat neurons, whereas the overproduction of STIM2 and Orai1 increased constitutive Ca^2+^ entry. Stronger interactions between STIM2 and Orai1 were observed in the absence of extracellular Ca^2+^ [13,14]. Similar observations were made by others using SOCE inhibitors or knocking down the main SOCE components [15,16]. SOCE has been implicated in various neuronal functions, such as neuronal plasticity [15,17], the expression and activity of PM receptors [18,19], neuronal excitability [6,20], and the regulation of gene expression [16]. Disruptions of SOCE have been implicated in several neurodegenerative diseases, including Alzheimer’s disease [17,21], Huntington’s disease [22,23], and epilepsy [6] (reviewed in [10,24]).

Notably, STIM2 appears to be crucial in regulating Ca^2+^ homeostasis in neurons. Reductions of both SOCE and basal Ca^2+^ levels were observed in cortical neurons that were isolated from *Stim2*^−/−^ mice [25]. STIM2 was also essential for SOCE in hippocampal neurons [17]. Descriptions of *Stim2*^−/−^ mice have not been fully consistent. Oh-Hora et al. reported early lethality in *Stim2*^−/−^ mice at 4-5 weeks of age [26]. Similarly, Berna-Erro et al. observed a lower life expectancy (i.e., 8 weeks) in *Stim2*^−/−^ mice [25]. The mechanism of death in *Stim2*^−/−^ mice remains unknown. However, another study showed no changes in mortality in *Stim2*^−/−^ mice [27]. Notably, however, *Stim* knockout in this work was restricted only to the forebrain. Despite a growing number of studies, the role of STIM2 in shaping behavior and neuronal activity remains elusive. In addition to the complexity of various processes that affect Ca^2+^ homeostasis in neurons, early lethality in mice that are devoid of STIM2 makes examining these aspects extremely challenging. The vast majority of studies have been performed in cell cultures, with a lack of such investigations in live organisms.

Zebrafish are a powerful model that allows combinations of behavioral tests, the in vivo imaging of neuronal activity, and molecular analyses. Studying zebrafish behavior provides vast possibilities even during the larval stage (reviewed in [28,29]). Assays that enable assessments of anxiety levels, such as thigmotaxis [30,31] and phototaxis [32,33], have been well established in this model. Zebrafish are also often used for drug screening, and several neuroactive compounds have been tested in this animal [28,34]. Among these compounds are pro-convulsant drugs, such as pentylenetetrazol (PTZ), that induce robust changes in larval behavior and neuronal activity [35].

In the present study, we took advantage of genome duplication that occurs in zebrafish. Through this process, zebrafish possess two genes that encode Stim2 proteins: *stim2a* and *stim2b* [3,4]. These genes share ~54% sequence identity (according to Ensembl release 99 [36]). The *stim2b* sequence is more similar to its human orthologue (~59% sequence identity) than the *stim2a* sequence (~49% sequence identity). Our group showed the presence of transcripts for both *stim2* genes and genes that encode other SOCE components in the adult zebrafish brain and head of zebrafish larvae [4]. Moreover, the mechanism of SOCE appears to be conserved in zebrafish and plays an important role in the development of this organism. The inhibition of this process disrupts cytokinesis in zebrafish embryos [37]. The downregulation of Stim1 in zebrafish resulted in disruptions of axonal growth and motor function [38]. Mutations of the *stim1* gene in zebrafish were also shown to affect the pigmentation process [39]. In the present study, we further characterized the phenotype of *stim2b^−/−^* zebrafish.

To unveil the function of Stim2b, we investigated the effects of Stim2b depletion on neuronal Ca^2+^ homeostasis and behavior. We investigated changes in gene expression that were induced by *stim2b* knockout. We performed an analysis of mutant locomotor activity using several behavioral tests. We also investigated the effects of treatment with drugs that modulate neuronal signaling on mobility. The behavioral analyses were supplemented with analyses of neuronal Ca^2+^ signaling in live zebrafish.

## 2. Materials and Methods

### 2.1. Animal Maintenance

Wild-type (WT; AB line), *stim2b^−/−^*, and Tg(*HuC:GCaMP5G*) [40] zebrafish were used in the study. All of the animals were maintained according to previously described methods [41] in the Zebrafish Core Facility that is a licensed breeding and research facility (PL14656251, registry of the District Veterinary Inspectorate in Warsaw; 064 and 051, registry of the Ministry of Science and Higher Education) at the International Institute of Molecular and Cell Biology in Warsaw. All of the experiments with larvae and adult fish were performed in accordance with the European Communities Council Directive (63/2010/EEC). Adult zebrafish and larvae were kept in E3 medium (2.48 mM NaCl, 0.09 mM KCl, 0.164 mM CaCl_2_·2H_2_O, and 0.428 mM MgCl_2_·6H_2_O) at 28.5 °C. Larvae were kept in a Petri dish (~50 larvae/dish) in an incubator under a 14 h/10 h light/dark cycle. The stages of fish development were defined as hours postfertilization (hpf) and days postfertilization (dpf).

### 2.2. Generation and Genotyping of stim2b^−/−^ Mutant Zebrafish

The clusters of regularly interspaced short palindromic repeats (CRISPR)/Cas9 system was applied to generate *stim2b^−/−^* zebrafish. CRISPR/Cas9 target sequences were selected using the web-based tool “CHOP CHOP” (https://chopchop.rc.fas.harvard.edu/; accessed September 15, 2014). The CRISPR/Cas9 target site (GGACCAGCACATCACGGTGG AGG) was localized in the exon 3 of *stim2b* (ENSDARG00000001776/ENDART00000012089). The templates for CRISPR guide RNA (gRNA) were prepared by polymerase chain reaction (PCR). The PCR fragments were column-purified using the QIAquick PCR Purification Kit (Qiagen, catalog no. 28106, Hilden, Germany) and transcribed using the mMESSAGE mMACHINE T7 Transcription Kit (Life Technologies, catalog no. AM1344M, Carlsbad, CA, USA) or the MEGAshortscript T7 Transcription Kit (Life Technologies, catalog no. AM1354, Carlsbad, CA, USA). The RNA was then column-purified with the miRNeasy Mini kit (Qiagen, catalog no. 217004, Hilden, Germany), and its quality was checked using an Agilent RNA 6000 Nano Chip (Agilent 2100 bioanalyzer, Santa Clara, CA, USA). A mixture that contained gRNA and Cas9 RNA (300 ng/µL; 1:1) was injected into one-cell-stage embryos (in a volume of 1 µL). The offspring of *stim2b*-targeted fish were screened using DNA sequencing of the targeted region for the presence of indel mutations. Fish with the insertion of 4 nucleotides were selected as P0 founders. F2 fish were outcrossed with the AB zebrafish line, and their offspring were in-crossed to generate homozygous mutants. The behavioral and real-time PCR (RT-PCR) and RNA sequencing (RNAseq) experiments were performed using the offspring of these fish (or fish from subsequent generations). For the Ca^2+^ imaging experiments, *stim2b^−/−^* fish were outcrossed with Tg(*HuC:GCaMP5G*) fish.

The offspring of P0 *stim2b^−/−^* founder fish were genotyped using high-resolution melting (HRM) analysis. Genetic material was obtained from the caudal fin of an adult fish, and the targeted region of *stim2b* was amplified using LightCycler 480 High-Resolution Melting Master (Roche, catalog no. 04909631001, Basel, Switzerland) and the LightCycler 96 System (Roche, Basel, Switzerland) or using Precision Melt Supermix (Bio-Rad, catalog no. 1725112, Hercules, CA) and the CFX Connect RT-PCR Detection System (Bio-Rad, Hercules, CA). The 4-nucleotide insertion caused a significant change in the PCR reaction product melting curve profile, allowing the identification of both homo- and heterozygotic mutants. Melting curve analysis was performed using LightCycler 96 SW 1.1 (Roche, Basel, Switzerland) or Precision Melt Analysis (Bio-Rad, Hercules, CA) software. The HRM results were confirmed by sequencing the PCR product, and these samples were used as standards in the subsequent experiments.

### 2.3. Drug Treatments

For drug treatments during the behavioral tests, 2× concentrated solutions were prepared in E3 medium. The 1.2 mM glutamate (Sigma-Aldrich, catalog no. G1251, Saint Louis, MO, USA) solution was prepared from 30 mM stock. The 3 and 30 mM PTZ (Sigma-Aldrich, catalog no. P6500, Saint Louis, MO, USA) solutions were prepared from 0.5 M stock. These solutions were mixed in a 1:1 proportion with the E3 medium in which the larvae were kept to obtain the final working doses of 600 µM glutamate and 1.5 or 15 mM PTZ.

### 2.4. Behavioral Experiments

Before the experiment, the larvae were kept in a Petri dish (~50 larvae/dish) in an incubator under a 14 h/10 h light/dark cycle. On the day of the experiment, randomly selected 4 dpf larvae were acclimated to the behavioral testing room for at least 30 min. Locomotor activity was recorded using the ZebraBox high-throughput monitoring system (ViewPoint Life Sciences, Lyon, France). The video files were further analyzed using EthoVision XT software (Noldus, Wageningen, the Netherlands). Data were exported to Microsoft Excel files and further analyzed using Excel (Microsoft, Redmond, WA, USA) and R software (R Foundation for Statistical Computing, Vienna, Austria, R package version 3.6.2). The results for larvae that were not active during the entire recording time (total distance <10 mm) were rejected. Adjusted values of *p* < 0.05 were considered statistically significant. The data are expressed as medians with first and third quartiles using boxplots, and dots represent data outliers unless otherwise stated. The numbers of fish that were used in each test are listed in Table 1.

#### 2.4.1. Open Field Test Adopted for Zebrafish Larvae

Two minutes before recording locomotor activity, the larvae were transferred to a 12-well plate that was then placed in the ZebraBox. The experiment was performed in a volume of 2 mL of E3 medium, and the light intensity was set to 70%. Locomotor activity was recorded for 10 min.

To analyze thigmotaxis, the area was divided into borders and a central area [30]. The experiment was divided into two 5 min periods. The time spent in each part of the well (s), mean total distance traveled (mm), mean velocity (mm/s), and frequency of fast movements were calculated and compared independently for these two time bins. Paired *t*-tests were used to compare these parameters between the border and central areas within groups. For comparisons between WT and *stim2b^−/−^* zebrafish, the Wilcoxon rank-sum test was used. Thigmotaxis was based on the time spent in each part of the well and calculated as the following: (duration of movement [in borders or center] + duration of no movement [in borders or center])/(duration of movement [total] + duration of no movement [total]) × 100%.

#### 2.4.2. Light Preference Test

This experiment was performed as described previously [33]. Two minutes before recording locomotor activity, the larvae were transferred to a Petri dish. Half of the dish was covered with two photographic filters (Cokin P154 ND8, Rungis, France), and the sidewall of this part of the dish was coated with black tape, which together blocked white light from the source above. The chamber was then placed in the ZebraBox. The experiment was performed in a volume of 20 mL of E3 medium, and the light intensity was set to 70%. Locomotor activity was recorded for 15 min.

The area was divided into dark and light parts. To assess phototaxis, the time spent in each part of the well (s) and mean total distance traveled (mm) in each part were calculated. Paired *t*-tests were used to compare these parameters between the dark and light parts within groups. For comparisons between WT and *stim2b^−/−^* zebrafish, the Wilcoxon rank-sum test was used. Phototaxis was assessed based on the time spent in the light part of the dish and calculated as the following: (duration of movement [light part] + duration of no movement [light part])/(duration of movement [total] + duration of no movement [total]) × 100%. Depending on this parameter, the responses of zebrafish were divided into three groups: phototaxis (>70% time in light part), no preference (>30% and <70% time in light part), and scototaxis (<30% time in light part). The distribution of these responses was compared between WT and *stim2b^−/−^* zebrafish using Pearson’s *χ^2^* test.

#### 2.4.3. Visual-Motor Response Test

This experiment was performed as described previously [42]. On the day before the experiment, the larvae were placed in 24-well plates that contained 1 mL of E3 medium. Thirty minutes before recording locomotor activity, the plates were placed in the ZebraBox. Two minutes before recording, half of the E3 medium volume was exchanged (based on treatment) for E3 medium, glutamate solution, or PTZ solution. The experiment consisted of three phases of the following changes in lighting conditions that were named according to long-lasting changes in zebrafish activity that are induced by these changes: baseline (0–10 min, 0% light intensity), low activity phase (10–20 min, 70% light intensity), and high activity phase (20–30 min, 0% light intensity). The mean total distance traveled (mm) and mean velocity (mm/s) were calculated independently for each of these phases. To compare the low and high activity phases, paired *t*-tests were used. For comparisons between treatments and genotypes, the Wilcoxon rank-sum test was used. For comparisons between more than two groups, the Kruskal–Wallis test was used, followed by the Wilcoxon–Mann–Whitney post hoc test with Benjamini and Hochberg correction. For PTZ treatment, we added to the analysis additional parameters, including the number of seizure-like episodes and amplitude of seizure-like episodes. Seizure-like episodes were defined as events of fast movement at a threshold of 200 mm/min, accompanied by whirlpool-like circular swimming. The number of these episodes was calculated during the entire duration of the experiment, and the amplitude of these episodes was equal to the distance traveled. The experiments were repeated three times.

### 2.5. In Vivo Imaging of Neuronal Activity

The in vivo imaging of Ca^2+^ signals in zebrafish neurons was performed using a Zeiss Lightsheet Z.1 microscope (Zeiss, Oberkochen, Germany, 40× objective, NA = 1.0). Zebrafish that expressed GCaMP5G under the neural promoter HuC were used in these experiments. Before the experiment, 4 dpf larvae were immobilized using the cholinergic blocker pancuronium bromide (0.6 µg/µL; Sigma-Aldrich, catalog no. P1918, Saint Louis, MO, USA) [43] and mounted in 1.5% low-melting-point agarose (Sigma-Aldrich, catalog no. A9414, Saint Louis, MO, USA). Time lapse images were recorded for 5 min at 28 °C (15 ms exposure, 1 frame/s). A single plane that contained the habenula, optic tectum, and cerebellum was selected. Oscillations of neuronal Ca^2+^ signals in the zebrafish larvae brain were observed. Using MATLAB (Mathworks, Natick, MA, USA), changes in fluorescence were extracted from the cells in the periventricular gray zone of the optic tectum (54–57 cells/fish) at single-cell resolution as described elsewhere [44]. Using a function that detects peaks based on changes in slope, peaks of oscillations of Ca^2+^ levels were selected. Such features as average oscillation frequency and the average amplitude of Ca^2+^ signals were quantified. The Wilcoxon rank-sum test was applied to compare mutant and WT zebrafish. Adjusted values of *p* < 0.05 were considered statistically significant. The data are expressed as medians with first and third quartiles using boxplots, and dots represent data outliers. The following number of cells were analyzed: WT (*n* = 456) and *stim2b^−/−^* (*n* = 336). The following number of animals were analyzed: WT (*n* = 8) and *stim2b^−/−^* (*n* = 6).

### 2.6. RNA Sequencing and Real-Time Polymerase Chain Reaction Expression Analysis

Total RNA was extracted from 5 dpf larvae using TRI Reagent (Invitrogen, catalog no. AM9738, Carlsbad, CA, USA) according to a previously published protocol [45]. Thirty larvae were pooled together to obtain one RNA sample. RNA quality was verified by measuring absorbance at 260, 280, and 230 nm. Only samples with absorbance >1.8 at A260/280 nm and A230/280 nm were used for further processing. Before RNAseq, RNA samples were additionally digested with DNase I and purified using the RNA Clean and Concentrator Kit (ZYMO Research, catalog no. R1013, Irvine, CA, USA) according to the manufacturer’s instructions. The sequencing procedure was performed using Illumina methodology. Preparation of the cDNA libraries and sequencing by Next-Generation Sequencing (Illumina, San Diego, CA, USA, NGS NextSeq 500) were performed in cooperation with the Zebrafish Core Facility at the International Institute of Molecular and Cell Biology. The sequencing resulted in approximately 40–70 million reads per sample with a 76 bp read length. The reads were extracted in FASTQ format and used for the subsequent analysis. The reads were then aligned to zebrafish RefSeq genome assembly annotated genes using the Ensembl annotation. The experiment was performed twice, and two independent samples from WT and *stim2b^−/−^* larvae were used in each experiment. Gene expression was calculated using the RNAseq by Expectation-Maximization (RSEM) method. Student’s *t*-test was used to compare fragments per kilobase per million mapped reads (FPKM)-normalized expression values between the WT and *stim2b^−/−^* groups. The *p* values were corrected for multiple testing. FASTQ files are available in the Sequence Read Archive (accession no. PRJNA627826).

For the RT-PCR analysis of expression, first-strand cDNA was synthesized using the SuperScript IV First-Strand Synthesis System (Invitrogen, catalog no. 18091050, Carlsbad, CA, USA) and 500 ng of RNA templates. RT-PCR was performed in duplicate using 25 ng of cDNA and FastStart Essential DNA Green Master (Roche, catalog no. 06402712001, Basel, Switzerland). The primers that were used for RT-PCR are listed in Table 2. Fold changes were calculated using the ^ΔΔ^Ct method. *Eukaryotic translation elongation factor 1 α1, like 1* (*eef1a1l1*) was used as a reference gene. Changes in expression are expressed as fold changes ± SD using expression in WT as a reference value. The calculations were performed in Microsoft Excel (Microsoft, Redmond, WA, USA).

## 3. Results

### 3.1. stim2b Knockout Does Not Induce a Severe Phenotype in Zebrafish Larvae but Affects Gene Expression and Neuronal Activity

**Figure 1:**
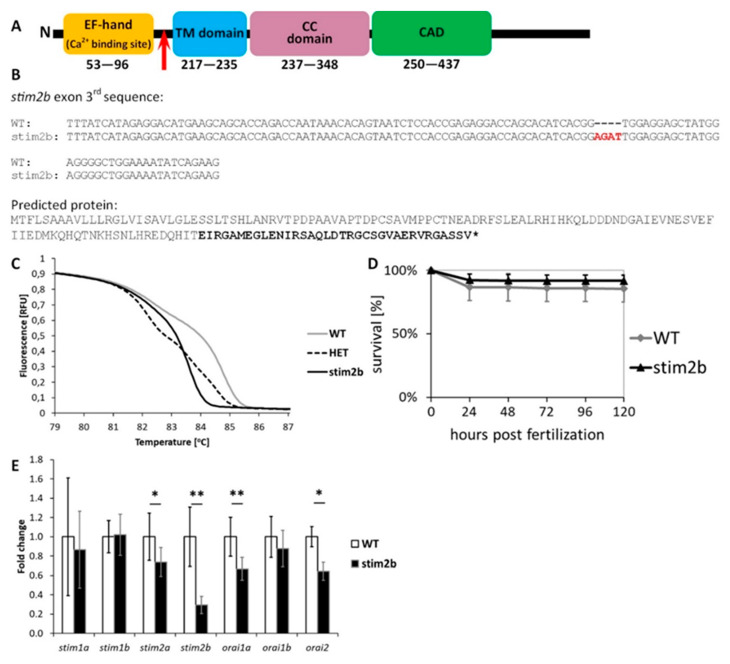
*stim2b^−/−^* mutant. (**A**) Schematic illustration of the most important domains of Stim2 protein. The location of the introduced mutation is marked with a red arrow. (**B**) DNA sequence of exon 3 of *stim2b* in wild-type (WT) and mutant zebrafish (upper) and predicted truncated protein sequence after introducing a frame-shift that cause the mutation (lower). (**C**) Representative results of the high-resolution melting (HRM) analysis that was used to discriminate mutant (stim2b), heterozygous (HET), and WT individuals. (**D**) Survival curve that shows the percentage of surviving WT and *stim2b^−/−^* zebrafish throughout the first 5 days of development. Number of experiments: 3, survival of 200 larvae of each genotype was estimated during each experiment. (**E**) RT-PCR results, presented as a mean fold change ± SD. * *p* < 0.05, ** *p* < 0.01, *** *p* < 0.001, mutant vs. WT (Student’s *t*-test). Number of experiments: 8.

We generated a *stim2b^−/−^* mutant zebrafish line using CRISPR/Cas9 technology. The presence of a frameshift mutation was confirmed by gDNA sequencing. The predicted protein was truncated at exon 3 before the transmembrane domain, which resulted in the disruption of interactions with membrane and cytoplasmic targets (Figure 1A,B). The presence of the mutation was confirmed by DNA sequencing and recognized as a shift in the melting curve by HRM analysis (Figure 1C). The development of homozygous *stim2b^−/−^* zebrafish did not show any irregularities. The viability of larvae between 6 hpf and 5 dpf was very similar to WT fish (Figure 1D). Adults were fertile and survived at least 20 months. As expected, a decrease in *stim2b* mRNA levels in the mutant zebrafish was observed. However, no compensatory response by other gene paralogues, such as *stim2a*, *stim1a*, or *stim1b*, occurred, but a slight decrease in *stim2a* expression was observed (Figure 1E). The downregulation of *orai1a* and *orai2*, which encode Orai1a and Orai2 channels that are involved in SOCE, was detected.

**Figure 2:**
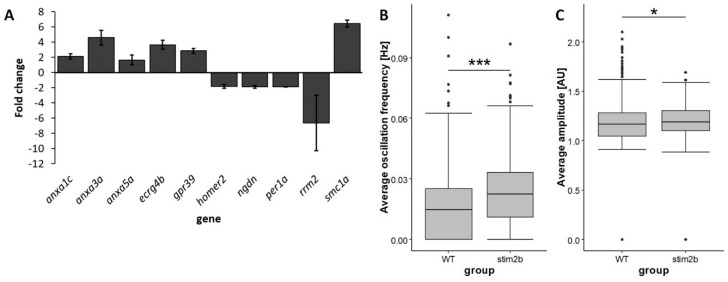
Differential expression of genes that are related to the nervous system and increases in the frequency and amplitude of neuronal Ca^2+^ oscillations in *stim2b^−/−^* larvae. (**A**) RNAseq results expressed as mean fold changes ± SD in *stim2b^−/−^* samples. The level of expression in WT was used as a reference value (= 1). Number of samples: 4. Number of experiments: 2. The exact fold change values and statistics are presented in Table 3. (**B**,**C**) Box plots of the average frequency (Hz) (**B**) and average amplitude (AU) (**c**) of Ca^2+^ signal oscillations in neurons in the periventricular gray zone of the optic tectum. * *p* < 0.05, ** *p* < 0.01, *** *p* < 0.001, mutant vs. WT (Wilcoxon rank-sum test). Number of cells: 456 (WT) and 336 (*stim2b^−/−^*). Number of animals: *n* = 8 WT, *n* = 6 *stim2b^−/−^*.

We next estimated changes in gene expression using RNAseq. A total of 96 genes were downregulated, and 180 were upregulated in *stim2b^−/−^* larvae compared with WT fish (Table S1). The RNAseq results are available in the Sequence Read Archive (accession no. PRJNA627826). We found several genes that are known to be expressed in the nervous system whose mRNA levels were altered in *stim2b^−/−^* mutants (Figure 2A, Table 3). Among these genes was *annexin3a* (*anxa3a*), which encodes a protein that is known to act as a Ca^2+^-dependent phospholipid-binding protein. Elevated levels of *annexin1c* (*anxa1c*) and *annexin5a* (*anxa5a*) were also observed. A few other genes whose products are known to be involved in neural development and function were differentially expressed. These included the upregulation of *ECRG4 augurin precursor b* (*ecrg4b*), *G protein-coupled receptor 39* (*gpr39*), and *structural maintenance of chromosomes 1A* (*smc1a*) and downregulation of *ribonucleotide reductase regulatory subunit M2* (*rrm2*), *neuroguidin* (*ngdn*), *period1a circadian clock* (*per1a*), and *homer2*. The *homer2* gene encodes a protein that is involved in several Ca^2+^ homeostasis processes in neurons, including the regulation of signaling via metabotropic glutamate receptors.

Next, we analyzed Ca^2+^ oscillations in neurons in *stim2b*^+/+^ and *stim2b^−/−^* larvae that expressed the GCaMP5G Ca^2+^ probe (Video S1, Video S2). The periventricular gray zone of the optic tectum was chosen as the region of interest. We observed a higher frequency of Ca^2+^ signal oscillations in *stim2b^−/−^* larvae (Figure 2B). Moreover, the amplitude of these signals was higher in *stim2b^−/−^* than in WT zebrafish (Figure 2C). Thus, we investigated whether these changes in expression patterns and neuronal activity correlated with alterations of *stim2b^−/−^* zebrafish behavior.

### 3.2. stim2b Knockout Increases Mobility and Thigmotaxis in Zebrafish Larvae

**Figure 3:**
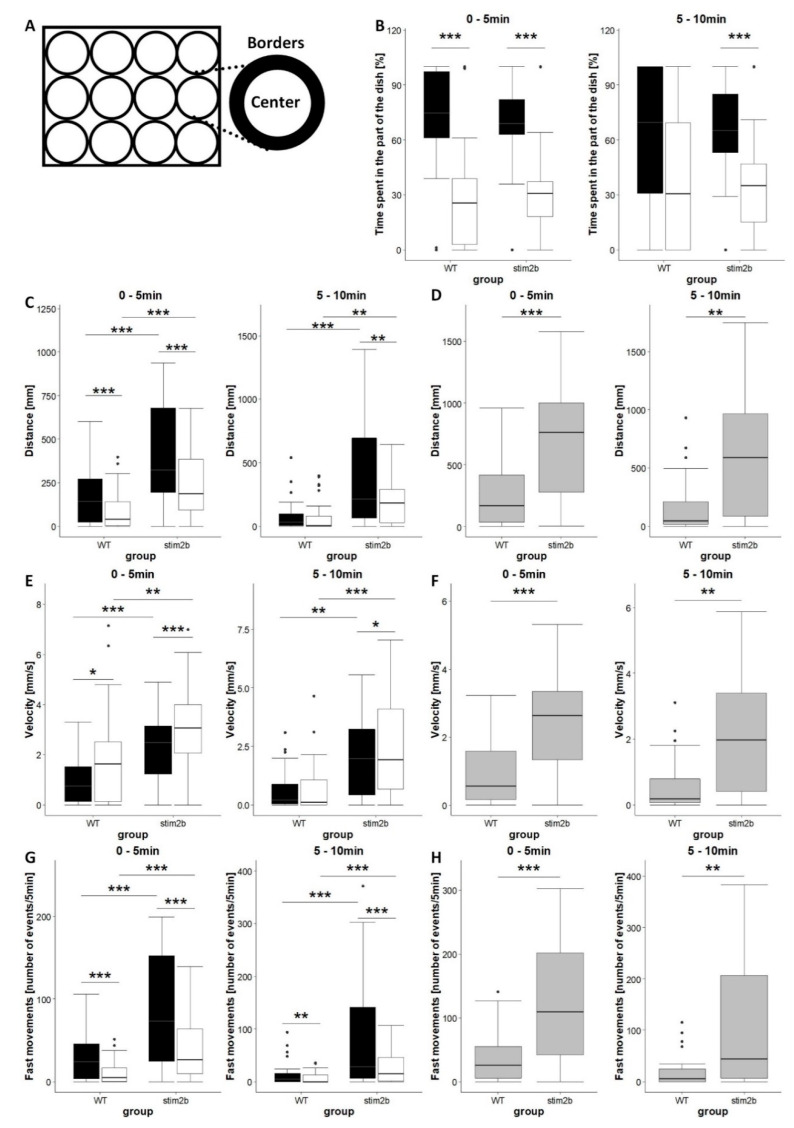
Increases in mobility and thigmotaxis in *stim2b^−/−^* larvae in the open field test. (**A**) Schematic diagram of the 12-well plates that were used in the experiments, showing a division into borders and central areas. (**B**,**C**,**E**,**G**) Boxplots of the time spent in each area (black in the border; white in center), distance traveled, velocity, and the frequency of fast movements in each part in WT and *stim2b^−/−^* larvae. (**D**,**F**,**H**) Boxplots of distance traveled, velocity, and the frequency of fast movements in WT and *stim2b^−/−^* larvae in both the border and central areas of the well combined. (**G**,**H**) Movements were considered fast movements when velocity was higher than 20 mm/s. * *p* < 0.05, ** *p* < 0.01, *** *p* < 0.001, border vs. center for each genotype (paired *t*-test) or mutant vs. WT (Wilcoxon rank-sum test). Number of larvae: *n* = 32 WT, *n* = 33 *stim2b^−/−^*. Number of experiments: 3.

We first checked basic parameters of locomotor behavior *in stim2b^−/−^* larvae. We analyzed zone preference according to a modified method of Schnörr et al. [30] (Figure 3A). This test allows analyses of general characteristics of zebrafish larvae mobility, such as distance traveled and velocity, and thigmotaxis. Zebrafish larvae have a tendency to stay close to borders of the well [31]. This behavior is closely related to anxiety. During the first 5 min, both WT and *stim2b^−/−^* fish spent most of the time in proximity to the borders of the well, with similar thigmotactic behavior between groups (Figure 3B, left). Larvae of both genotypes traveled longer distances along the borders of the well (Figure 3C, left) but moved with higher velocity in the central area (Figure 3D, left). After the initial acclimatization period, WT zebrafish began to spend more time in the central area, whereas *stim2b^−/−^* zebrafish did not alter their behavior and still moved close to the borders of the well (Figure 3B, right). Mutant larvae also continued to travel a longer distance along the borders of the well and moved with a higher velocity in the central area, in contrast to WT zebrafish (Figure 3C,D, right).

All of the analyzed parameters indicated that *stim2b^−/−^* zebrafish generally exhibited higher mobility than WT zebrafish (Figure 3C–H). Mutant zebrafish covered a longer distance (Figure 3C,D), moved with a higher velocity (Figure 3E,F), and more frequently exhibited high-speed movements (Figure 3G,H) both along the borders and in the central area during both phases of the experiment.

### 3.3. stim2b^−/−^ Mutants Exhibited Disruptions of Phototaxis but a Normal Visual-Motor Response

**Figure 4:**
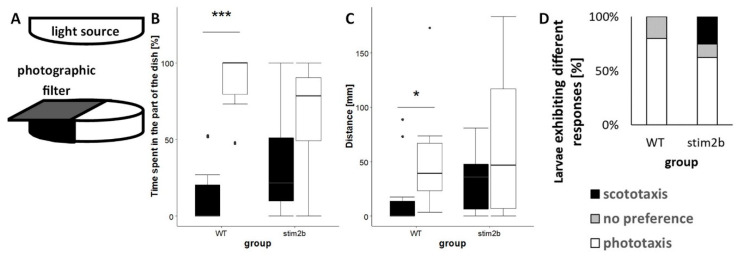
Disruption of phototaxis in *stim2b^−/−^* larvae in the light preference test. (**A**) Schematic diagram of the experimental design. The Petri dish was exposed to white light that emanated from above the dish. Illumination of half of the area of the dish was limited using a gray photographic filter. (**B**,**C**) Boxplots of the time spent and distance traveled in each part of the dish (black in the dark part, white in the light part) in WT and *stim2b^−/−^* larvae. * *p* < 0.05, ** *p* < 0.01, *** *p* < 0.001, dark vs. light part for each genotype (paired *t*-test). (**D**) Stacked bar chart of the distribution of different types of responses: phototaxis (>70% time in the light part), no preference (30-70% time in the light part), and scototaxis (<30% time in the light part). The distribution of responses was compared using Pearson’s *χ^2^* test (*p* = 0.2419). Number of larvae: *n* = 10 WT, *n* = 8 *stim2b^−/−^*. Number of experiments: 5.

To further investigate anxiety-like behavior in *stim2b^−/−^* zebrafish, the light preference test was performed as described previously [32,33] (Figure 4A). Zebrafish larvae exhibited a tendency to stay in the light part and actively avoid the dark part [30]. As expected, WT larvae spent significantly more time and covered a longer distance in the light part than in the dark part of the well (Figure 4B, C). In contrast, *stim2b^−/−^* zebrafish exhibited no significant preference for either the light or dark part. The majority of WT larvae (80%) exhibited clear phototaxis, preferring the light part of the well, and 20% had no preference (Figure 4D). Only 60% of the mutant fish exhibited phototaxis, 15% had no preference, and 25% exhibited scototaxis (i.e., preference for dark). The latter behavior was not observed in any WT larvae.

**Figure 5:**
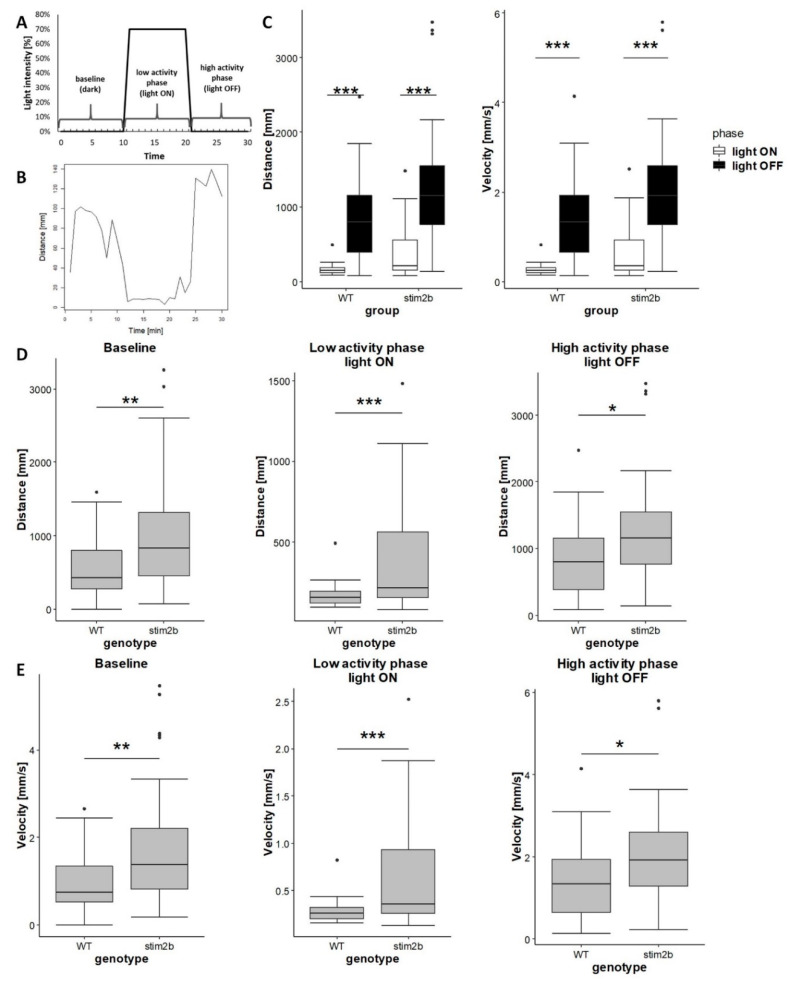
Light perception was unaffected in *stim2b^−/−^* larvae in the visual-motor response test, indicating hyperactivity of these mutants. (**A**) Experimental design that shows changes in light intensity during the experiment. Larva activity was recorded during a 30 min period that consisted of a baseline phase (0% light), inactive phase (70% light), and high activity phase (0% light). (**B**) Representative plot of the distance traveled by a single larva during 1 min of the experiment. The plot shows the response of WT larvae. (**C**) Boxplots of the distance traveled and velocity in WT and *stim2b^−/−^* larvae after turning the light on (in white) and off (in black). * *p* < 0.05, ** *p* < 0.01, *** *p* < 0.001, response to turning the light on vs. off for each genotype (paired *t*-test). (**D**) Boxplots of the distance traveled and (**E**) velocity in WT and *stim2b^−/−^* larvae during each phase of the experiment. * *p* < 0.05, ** *p* < 0.01, *** *p* < 0.001, mutant vs. WT (Wilcoxon rank-sum test). Number of larvae: *n* = 36 WT, *n* = 35 *stim2b^−/−^*. Number of experiments: 3.

To exclude the possibility that disruptions of phototaxis reflected vision deficits, we tested motor responses to sudden changes in light in *stim2b^−/−^* larvae using a modified protocol that was described by Liu et al. [42] (Figure 5A). Such a stimulus evokes an immediate startle response in zebrafish larvae, followed by a long-lasting change in activity [46]. As expected, when the light was turned on, WT fish reacted with a startle response, followed by a decrease in mobility (i.e., low activity phase). When the light was turned off, they increased their mobility (i.e., high activity phase; Figure 5B,C). The same response was observed in *stim2b^−/−^* larvae, indicating that they had a similar ability to differentiate between dark and light as WT fish. Thus, this visual-motor response test excluded the possibility that abnormal phototaxis in *stim2b^−/−^* mutants resulted from a disruption of light perception. Despite the similar behavior in response to the light stimulus, *stim2b^−/−^* larvae exhibited higher activity than WT larvae during all phases of the experiments (Figure 5D,E). These data confirmed that *stim2b^−/−^* mutants were hyperactive.

### 3.4. Response to GABAergic and Glutamatergic Signaling Modulators is Affected by Stim2b Knockout

**Figure 6:**
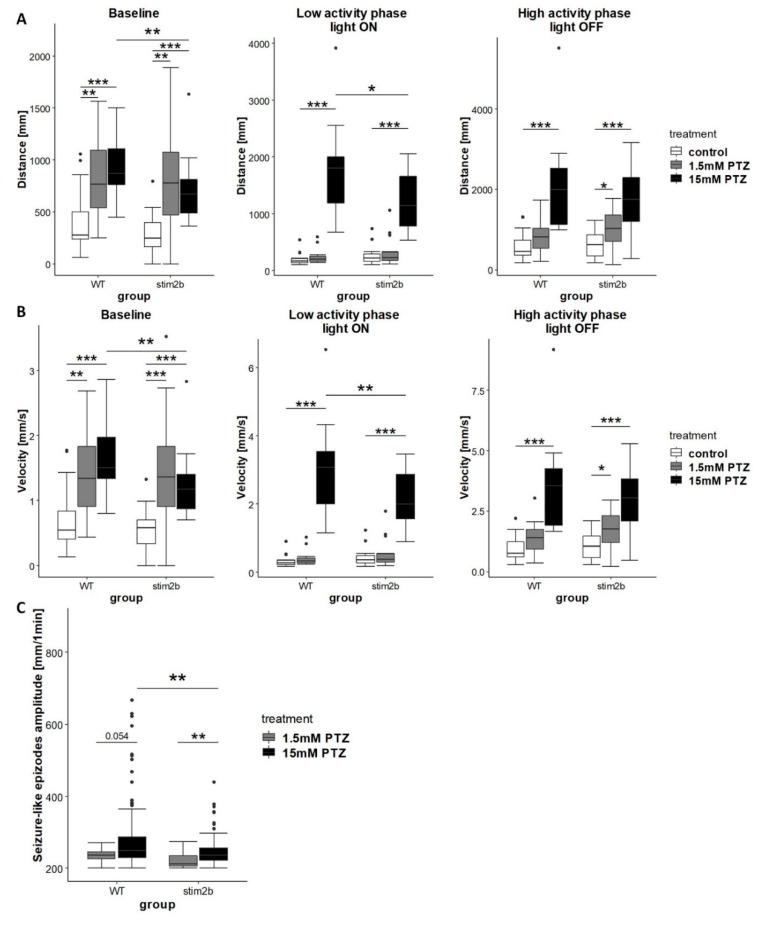
Treatment with the low dose of pentylenetetrazol (PTZ) induced stronger reaction of *stim2b^−/−^* than WT larvae and treatment with high dose of PTZ induce weaker reaction of *stim2b^−/−^* than WT larvae. The experimental design was the same as described in Figure 5A. Larva activity was recorded during a 30 min period that consisted of a baseline phase (0% light), low activity phase (70% light), and high activity phase (0% light). Before the experiment, half of the medium in the dish was exchanged with PTZ solution (final concentration: 1.5 or 15 mM). (**A**,**B**) Boxplots of the distance traveled (**A**) and velocity (**B**) in WT and *stim2b^−/−^* larvae during all phases of the experiment. (**C**) Boxplots of the average amplitude (distance traveled during 1 min) of seizure-like episodes that occurred during the experiment in WT and *stim2b^−/−^* larvae. Seizure-like episodes were defined as events of a long distance traveled within a short period of time (>200 mm within 1 min). * *p* < 0.05, ** *p* < 0.01, *** *p* < 0.001, PTZ-treated larvae vs. control group of the same genotype (Wilcoxon–Mann–Whitney *post hoc* test with Benjamini and Hochberg correction) or mutant vs. WT with the same treatment (Wilcoxon rank-sum test). Number of larvae: *n* = 18 for all groups, with the exception of *n* = 14 WT that were treated with 1.5 mM PTZ. Number of experiments: 3.

The increase in mobility, the presence of circling behavior, and neuronal hyperactivity in *stim2b^−/−^* mutants suggested the presence of seizure-like activity. We next investigated the response of *stim2b^−/−^* larvae to PTZ, a drug that is used to induce seizures. We expected that blocking inhibitory γ-aminobutyric acid (GABA)ergic signaling with PTZ would reveal greater susceptibility to seizures in mutant fish. Using the visual-motor response test (described in Section 3.3), we first tested different doses of PTZ. We found that 1.5 mM PTZ had a weak effect, whereas 15 mM PTZ significantly increased activity and induced the presence of rapid “whirlpool-like” circular swimming behavior in both WT and *stim2b^−/−^* larvae (Figure 6). Upon stimulation with the lower dose of PTZ, WT larvae exhibited an increase in mobility only during the first 10 min of the test, whereas *stim2b^−/−^* larvae traveled a longer distance and had a higher velocity also during high activity phase (Figure 6A,B). The response to treatment with the high dose of PTZ was lower in *stim2b^−/−^* mutants than in WT larvae. Both WT and *stim2b^−/−^* larvae, increased their mobility upon 15mM PTZ treatment, however during the baseline and low activity phases caused *stim2b^−/−^* larvae to move a shorter distance and have a lower velocity than WT larvae (Figure 6A,B). We analyzed the occurrence of seizure-like episodes, which were characterized by a high velocity and long distance traveled. The mean amplitude of seizure-like episodes, defined as the distance traveled, was higher in WT fish (Figure 6C). These results showed that *stim2b^−/−^* larvae exhibited greater susceptibility to seizures with a low dose of PTZ, but they exhibited a weaker response to the high dose of PTZ.

**Figure 7:**
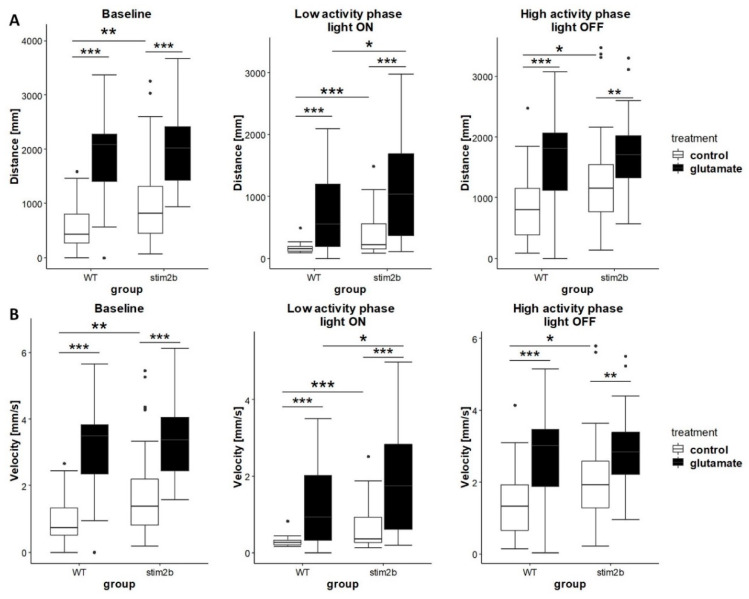
Glutamate treatment induced a stronger response in *stim2b^−/−^* mutants than in WT larvae. The experimental design was the same as described in Figure 5A. Activity was recorded during a 30 min period that consisted of a baseline phase (0% light), low activity phase (70% light), and high activity phase (0% light). Before the experiment, half of the medium in the dish was exchanged with a glutamate solution (final concentration: 600 µM). (**A**,**B**) Boxplots of the distance traveled (**A**) and velocity (**B**) in WT and *stim2b^−/−^* larvae during all phases of the experiment. * *p* < 0.05, ** *p* < 0.01, *** *p* < 0.001, glutamate-treated larvae vs. control group of the same genotype or mutant vs. WT (Wilcoxon rank-sum test). Number of larvae: *n* = 36 WT, *n* = 35 *stim2b^−/−^*. Number of experiments: 3.

We next investigated the response of mutant and WT zebrafish to glutamate. Glutamate signaling plays an important role in epileptogenesis [47], and STIM proteins were shown to affect glutamatergic transmission [19,48,49]. We used the same protocol as for PTZ treatment to investigate the effect of glutamate on mobility in the low and high activity phases. Both WT and mutant zebrafish that were treated with 600 µM glutamate exhibited a significant increase in activity, reflected by a longer distance traveled and higher velocity (Figure 7). When the light was turned on (i.e., low activity phase), *stim2b^−/−^* mutants that were treated with glutamate traveled a longer distance and had a higher velocity compared with WT zebrafish. When the light was turned off (i.e., high activity phase), the distance traveled and velocity in *stim2b^−/−^* larvae that were treated with glutamate did not differ from WT larvae. In summary, a stronger response to glutamate was observed in *stim2b^−/−^* mutants only under conditions of sudden light exposure immediately after the startle response. This suggests that higher activity in stim2b^−/−^ mutants might be attributable to the dysregulation of neuronal Ca^2+^ homeostasis.

## 4. Discussion

STIM2 protein is essential for SOCE in hippocampal neurons [17]. In cortical neurons that were isolated from *Stim2*^−/−^ mice, basal Ca^2+^ levels and SOCE decreased [25]. However, the role of STIM2 in shaping behavior remains unclear. Early lethality in mice that lack STIM2 [25,26] makes such studies challenging. To address this problem, we took advantage of zebrafish genetics, which possess two genes that encode Stim2 (*stim2a* and *stim2b*). We created a zebrafish line with *stim2b* knockout, which appeared to be viable and fertile while having only a weak neurological phenotype. In these mutant zebrafish, we detected the significant downregulation of *stim2a*, *orai1a*, and *orai2* and non-sense-mediated decay of the *stim2b* transcript. Thus, we cannot exclude the possibility that the observed phenotypes (e.g., increase in thigmotaxis, disruption of phototaxis, differential responses to PTZ and glutamate, a higher frequency of Ca^2+^ spikes, and a higher amplitude of Ca^2+^ spikes) resulted from the dysregulation of SOCE rather than a deficiency of Stim2b itself. However, the presence of functional Stim2a in the mutant fish might obscure the effect of *stim2b* knockout. In addition to changes in the levels of mRNA of SOCE components, we found the differential expression of several genes that encode proteins that are important for the function and development of the nervous system. The differential expression of these genes might be responsible for the observed behavioral changes in mutant zebrafish.

One of the upregulated genes was *anxa3a*, the levels of which were 4.5-times higher in mutant larvae than in WT larvae. Additionally, *anxa1c* and *anxa5a* expression was slightly higher in *stim2b^−/−^* larvae. Notably, *annexin*s upregulation was observed in epileptic brains [50,51]. A significant increase in ANXA3 levels was also found in brain injuries [52,53]. Expression of the *ecrg4b* gene, which encodes Augurin protein, was also elevated. Augurin is involved in nervous system development [54] and was shown to be upregulated in brain samples that were isolated from aged mice [55] and mice that overexpressed Tau [56]. Another gene that was upregulated in mutant larvae was *gpr39*. This gene belongs to the ghrelin receptor family and encodes a G-protein-coupled receptor that senses changes in extracellular zinc ion levels. Signaling via GPR39 may reduce excitatory activity during seizure-related excessive neuronal firing that is caused by enhanced GABAergic responses [57]. The higher level of *gpr39* expression that was observed in the present study may affect inhibitory signaling in *stim2b^−/−^* larvae and protect against excessive neuronal activity that is induced by high-dose PTZ stimulation. GPR39 was also shown to modulate anxiety-like behavior, and GPR39 expression was increased by antidepressant administration [58]. A very high six-fold increase in *smc1a* expression was observed, which encodes a member of the cohesion complex that is responsible for chromosome segregation during cell division. The downregulation of *smc1a* impaired neuronal development in zebrafish [59], and mutations of this gene were found in patients with epilepsy [60,61].

Four genes that were downregulated are potentially interesting: *rrm2*, *ngdn*, *per1a*, and *homer2*. Rrm2 is involved in DNA replication and was shown to be crucial for proper development of the nervous system in zebrafish. A mutation of *rrm2* resulted in the disorganization of forebrain glia and severe axonal pathfinding errors in zebrafish larvae [62]. The *ngdn* gene encodes a translational regulatory protein that is active during development of the vertebrate nervous system and plays a role in synaptic plasticity [63]. The *per1a* gene is involved in photoperiodism. Interestingly, the downregulation of its paralogue, *per1b*, resulted in an increase in motor activity in zebrafish larvae and aberrant development of the dopaminergic system [64]. The *homer2* gene encodes a protein that is involved in synaptic plasticity and regulates neurotransmission [65]. It is involved in Ca^2+^ homeostasis by regulating the activity of proteins that are involved in Ca^2+^ signaling, including metabotropic glutamate receptors, inositol triphosphate receptors, and transient receptor potential channels [66,67]. It also participates in regulating basal cytosolic Ca^2+^ via an interaction with the plasma membrane calcium reuptake pump [68]. The decrease in Homer2 levels appeared to be responsible for the observed changes in the response to glutamate stimulation in *stim2b^−/−^* larvae.

Only a few studies have investigated the role of STIM2 in behavior, but the results have been inconclusive. Mice that overexpressed STIM2 and ORAI1 in neurons exhibited reductions of anxiety-like behavior, including increases in exploration of the arena in the open field test and time spent on the open arms of the elevated plus maze [69]. Mice with double *Stim1*/*Stim2* conditional knockout in the forebrain spent more time on the open arms of the elevated plus maze, suggesting a greater propensity to engage in exploration and a decrease in anxiety-like behavior [27], thus *Stim1/Stim2* knockout in mice gave a similar effect as STIM2 and ORAI1 overexpression. In the present study, zebrafish larvae that were devoid of Stim2b exhibited an increase in mobility after they were transferred to a new dish, and thigmotactic behavior lasted longer in mutant larvae than in WT larvae. Thigmotaxis is considered to be related to anxiety [28,31]. Therefore, the results of the thigmotaxis test indicated an increase in anxiety-like behavior in *stim2b^−/−^* zebrafish. To investigate whether *stim2b^−/−^* mutants exhibited an anxiety-related phenotype, we performed the light preference test. The tendency of zebrafish larvae to avoid darkness is positively correlated with their anxiety level [28]. This test showed the disruption of phototaxis in *stim2b^−/−^* zebrafish and also an opposite tendency (i.e., scototaxis) in some fish. Thus, contrary to the results of the open field test, this observation suggested a decrease in anxiety-like behavior. The visual-motor response test was performed [42] to exclude the possibility that *stim2b^−/−^* mutants had difficulties in visually distinguishing between dark and light. The results indicated that the disrupted phototaxis in *stim2b^−/−^* larvae was not attributable to visual impairments. Moreover, the visual-motor response test showed that *stim2b^−/−^* larvae were hyperactive during all phases of the test, independent of the lighting conditions. Similar hyperactivity was observed in zebrafish larvae in models of attention-deficit/hyperactivity disorder (ADHD), such as *micall2b* knockout [70] and *lphn3.1* knockdown [71]. Notably, variations of STIM2 copy number were observed in ADHD patients [72], and deficits in learning ability that are observed in *Stim2*^−/−^ mice [25] are also a feature of ADHD.

An increase in thigmotaxis might be related not only to an increase in anxiety, but it also can be an indicator of circling behavior. In a previous study, PTZ-treated larvae exhibited an increase in mobility and swimming abnormalities, characterized by circular trajectories [35]. Disruptions of phototaxis may also be a sign of circling behavior in *stim2b^−/−^* larvae, which does not depend on light. Thus, the changes in behavior that were observed in *stim2b^−/−^* mutants in the present study may be related to seizure-like activity. A similar phenotype (i.e., increase in thigmotaxis and decrease in phototaxis) was also previously observed in *tsc* mutant larvae, which had greater susceptibility to PTZ-induced epileptogenesis [33]. Larvae with *depdc5* knockdown exhibited hyperactivity early in development (28 hpf) and aberrant locomotion with an increase in circular swimming (3 dpf) that was correlated with neuronal hyperactivity [73].

We also found that *stim2b^−/−^* larvae exhibited a greater frequency and amplitude of Ca^2+^ oscillations in the periventricular zone of the optic tectum. This structure is involved in processing visual stimuli and the activation of motor responses. Therefore, disruption of the phototactic response may be attributable to an increase in activity in this region. Disruption of the phototactic response in *stim2b^−/−^* mutant zebrafish and their increase in mobility may have occurred because their neuronal network did not properly develop. This defect may result from the dysregulation of genes that are important for neurodevelopment, such as *rrm2*, *ngdn*, and *smc1a*. Notably, an increase in neuronal activity upon seizure induction was observed in the optic tectum in zebrafish [74]. Therefore, abnormal behavior in *stim2b^−/−^* larvae, combined with neuronal hyperactivity and the upregulation of annexin gene expression, suggested seizure-like activity in these mutants.

Greater susceptibility to PTZ treatment is observed in genetic zebrafish models of epilepsy. Therefore, we investigated the response of *stim2b^−/−^* larvae to this drug. We found that *stim2b^−/−^* larvae responded to the low dose of PTZ with a higher distance traveled and velocity in the high activity phase of the visual-motor response test. In contrast, *stim2b^−/−^* larvae that were treated with the high dose of PTZ reacted with a shorter distance traveled and velocity compared with WT zebrafish in the low activity phase of the test. These results indicated the distinct sensitivity of mutant larvae to PTZ compared with WT zebrafish, thus further indicating that the mutants exhibited seizure-like activity.

To further unveil the possible mechanism of behavioral alterations in *stim2b^−/−^* larvae, we observed their response to glutamate stimulation. On the basis of the available literature we could suggest that the glutamate is able to reach neurons in zebrafish larvae [75]. However, the effect of glutamate treatment can be a result of stimulation taste receptors. Upon glutamate treatment, we observed an increase in mobility in both WT and mutant zebrafish, but *stim2b^−/−^* mutants were more active during the inactive phase of the visual-motor response test. Changes in *gpr39* and *homer2* levels can affect neuronal excitability and may contribute to hyperactivity of *stim2b^−/−^* larvae and greater sensitivity to PTZ and glutamate treatment. STIM2 has been shown to have complex actions on glutamate signaling. STIM2 was shown to increase glutamate signaling via α-amino-3-hydroxy-5-methyl-4-isoxazolepropionic acid receptors [19,48]. The downregulation of STIM2 enhanced Ca^2+^ influx via NMDA receptors [49]. Therefore, the higher responsiveness of *stim2b^−/−^* zebrafish may be related to an increase in NMDA receptor activity. However, the effects of STIM2 on voltage-gated calcium channels cannot be excluded, although no such data are available. STIM1 was shown to affect the activity of voltage-gated calcium channels through interactions with the SOAR domain that shares ~82% sequence identity with STIM2 and 84% sequence identity with the zebrafish Stim2b SOAR (according to Ensembl release 99 [36]).

## 5. Conclusions

We created *stim2b^−/−^* zebrafish that did not present a severe phenotype or lower viability. We speculate that the dysregulation of SOCE affects gene expression. Such effects have been previously described (reviewed in [9]). We observed the differential expression of several genes in *stim2b^−/−^* larvae. Among these genes were *rmm2*, *ngdn*, and *smc1a*, whose aberrant levels may affect the development of brain structures in *stim2b^−/−^* larvae. Moreover, neuronal activity in these mutants was affected, with a higher frequency and amplitude of Ca^2+^ oscillations than in WT larvae. The behavioral experiments indicated an increase in mobility and the occurrence of circling behavior in *stim2b^−/−^* zebrafish. Our findings suggest that *stim2b*^−/−^ larvae exhibited seizure-like activity, which was confirmed by their stronger responses to treatment with glutamate and a low dose of PTZ. However, the mechanism that links Stim2b with the higher frequency of neuronal Ca^2+^ oscillations and behavioral changes (e.g., hyperactivity and seizure susceptibility) is unclear and requires further investigation.

## Figures and Tables

**Table 1:** Summary of numbers of animals and replications of experiments for all behavioral tests.

Type of Test	Genotype	Treatment	Number of Larvae	Number of Experiments
Open field test adopted for zebrafish larvae	WT	E3	32	3
	*stim2b^−/−^*		33	
Light preference test	WT	E3	10	5
	*stim2b^−/−^*		8	
Visual-motor response test–no treatment	WT	E3	36	3
	*stim2b^−/−^*		35	
Visual-motor response test–glutamate treatment	WT	600 µM glutamate	36	3
	*stim2b^−/−^*		35	
Visual-motor response test–PTZ treatment	WT	1.5 mM PTZ	14	3
	*stim2b^−/−^*		18	
	WT	15 mM PTZ	18	
	*stim2b^−/−^*		18	

**Table 2:** Primers that were used for Real-Time Polymerase Chain Reaction.

Gene	Forward Primer	Reverse Primer
*eef1a1l1*	AAAATCGGTGGTGCTGGCAA	GGAACGGTGTGATTGAGGGA
*stim1a*	TGAATTCGGATTGCCAGTCGT	TTCAAGTCCCTCTGCGAACC
*stim1b*	TGAGTTTTGAGGCCATCCGC	AACCCATCCGTCTCTGTCAC
*stim2a*	ATTACGGAGGCGGATCGATT	CCTCAATGCCTCCATCCTGA
*stim2b*	CTGGTGGAGTGGACGATCTT	CGTCAGAGGAGGTCGAATCA
*orai1a*	GTGCATTTTTACCGCTCGCT	TTGAAGAGGCATCTCCCCTC
*orai1b*	GCTGTAAGCAACGTGCACAA	TCCCGATGACGGTGGAAAAG
*orai2*	CGAGCTAGCCTGGGGTTTTT	AGTCAACCGGCAGGAACTTG

**Table 3:** RNAseq results of the differential expression of genes that are related to the nervous system in *stim2b^−/−^* larvae. Student’s *t*-test was used to compare FPKM-normalized expression values between mutant and WT, and the *p* values were corrected for multiple testing. * *p* < 0.05 (FDR < 0.05), ** *p* < 0.01 (FDR < 0.05), significant difference in fold changes. Number of samples: 4. Number of experiments: 2.

Gene	Mean Fold Change	*p*	False Discovery Rate
*anxa1c*	2.13	0.0027	0.0874
*anxa3a*	4.59 *	0.0002	0.0332
*anxa5a*	1.64	0.0083	0.1387
*ecrg4b*	3.65 *	0.0001	0.0271
*gpr39*	2.87 *	0.0003	0.0380
*homer2*	0.54 *	0.0004	0.0426
*ngdn*	0.54 *	0.0004	0.0426
*per1a*	0.54 **	<0.0001	0.0066
*rrm2*	0.15 *	0.0007	0.0500
*smc1a*	6.45 *	<0.0001	0.0142

## Supplementary Materials

The following are available online at https://www.mdpi.com/2073-4409/9/5/1285/s1, Table S1: RNAseq results of the differential expression of genes in *stim2b^−/−^* larvae. Video S1: Ca^2+^ oscillations in neurons of *stim2b*^+/+^. Video S2: Ca^2+^ oscillations in neurons of *stim2b^−/−^*.
